# Mechanisms of action of anti-inflammatory proteins and peptides with anti-TNF-alpha activity and their effects on the intestinal barrier: A systematic review

**DOI:** 10.1371/journal.pone.0270749

**Published:** 2022-08-08

**Authors:** Mayara Santa Rosa Lima, Vanessa Cristina Oliveira de Lima, Grasiela Piuvezam, Kesley Pablo Morais de Azevedo, Bruna Leal Lima Maciel, Ana Heloneida de Araújo Morais

**Affiliations:** 1 Biochemistry and Molecular Biology Postgraduate Program, Biosciences Center, Federal University of Rio Grande do Norte, Natal, RN, Brazil; 2 Public Health Postgraduate Program, Center for Health Sciences, Federal University of Rio Grande do Norte, Natal, RN, Brazil; 3 Department of Public Health, Federal University of Rio Grande do Norte, Natal, RN, Brazil; 4 Nutrition Postgraduate Program, Center for Health Sciences, Federal University of Rio Grande do Norte, Natal, RN, Brazil; 5 Department of Nutrition, Federal University of Rio Grande do Norte, Natal, RN, Brazil; USDA-Agricultural Research Service, UNITED STATES

## Abstract

Several studies in animal models of intestinal inflammation have been performed with the aim of understanding the mechanisms of action of anti-inflammatory proteins and peptides that reduce TNF-α. In order to present the best targets, effects and strategies for the treatment of intestinal inflammation in experimental models, this systematic review (SR) aimed to answer the following question: what are the mechanisms of action of molecules with anti-TNF-α activity on the intestinal barrier? The SR protocol was registered in the International Prospective Register of Systematic Reviews (PROSPERO, number CRD42019131862) and guided by the methodological procedures used for the elaboration of the SR. Articles that were part of the SR were selected considering the eligibility criteria according to the PICO (Population, Intervention, Comparison/Control and Outcomes) and were searched in the PubMed, Scopus, Web of Science, Excerpta Medica Database (EMBASE) and ScienceDirect databases. Twenty-five articles reporting studies in rats and mice were selected and the risk of bias was assessed using the tool from the SYstematic Review Center for Laboratory Animal Experimentation (SYRCLE). A descriptive synthesis of the results obtained was carried out. Based on the results, the anti-inflammatory molecules that reduced TNF-α acted mainly on the TNF-TNFR1/TNFR2 and TLR4/MD2 complex signaling pathways, and consequently on the NF-κB pathway. This improved the aspects of the inflammatory diseases studied. In addition, these mechanisms also improved the macroscopic, histological and permeability aspects in the intestine of the animals. These findings point to the potential of protein and peptide molecules that act on inflammatory pathways for medical applications with specific and promising strategic targets, aiming to improve inflammatory diseases that affect the intestine. This systematic review also highlights the need for more details during the methodological description of preclinical studies, since this was a limitation found.

## Introduction

In addition to being involved in food processing and digestion, the gastrointestinal tract is a space for dynamic interactions between host cells and the external environment. Together with the chemical barrier and the cellular immune system, the epithelial cell layer plays an essential role as the first barrier against external factors and maintains a symbiotic relationship with commensal bacteria. Therefore, an imbalance in the structure of this barrier can trigger an immune reaction and uncontrolled microbial growth and is related to inflammatory bowel disorders, extra-intestinal autoimmune diseases, obesity and metabolic disorders [1].

Intestinal homeostasis is maintained through the strict balance between cell death and proliferation to prevent intestinal inflammation and tumor formation. In the regulation of this balance, tumor necrosis factor-alpha (TNF-α) is a central inflammatory cytokine that plays a crucial role [2].

TNF-α modulates multiple signaling pathways, being vital in the immune response, regulating the immediate inflammatory reaction with innate immune involvement, and cell activation with subsequent proliferation and programmed cell death or necrosis. However, because of this broad spectrum of cellular effects, TNF-α is also implicated in various disease states, such as inflammatory bowel diseases, infectious diseases, intestinal wound healing and tumor formation [3].

Due to the participation of TNF-α in the pathogenesis of several inflammatory disorders, anti-TNF-α molecules have been used over the past few years to treat inflammatory bowel diseases and other conditions that include high concentrations of this cytokine, such as rheumatoid arthritis and psoriasis [4], although the anti-TNF-α therapy is associated with worsening multiple sclerosis, another inflammatory disease [5].

Anti-TNF-α therapy consists of the use of antibodies that act to neutralize soluble TNF-α, but each agent has specific actions due to its distinct pharmacological properties [6].

The side effects and risk of loss of effect of classic anti-TNF-α therapy, however, have stimulated the search for alternatives for anti-inflammatory treatment, with the development of new classes of antibodies [7], including those that act on other cytokines or components involved in TNF-α signaling pathways, culminating in its reduction. In addition, other classes of proteins and also bioactive peptides have been studied as treatment strategies that reduce inflammation and improve the integrity and function of the intestinal barrier [8, 9].

Several preclinical studies with models of intestinal inflammation aimed to understand the mechanisms of action of these anti-inflammatory proteins and peptides that reduce TNF-α. Such studies describe the mechanisms involved in the action of these molecules to develop the best strategies for treating these diseases [10–12]. Therefore, this systematic review aimed to study the mechanisms of action of these proteins and peptides and their effects on the inflamed intestinal barrier in animal models. Thus, the starting question was: what are the mechanisms of action of molecules with anti-TNF-α activity on the intestinal barrier?

## Methodology

The protocol of this systematic review (SR) was prepared following the guidelines described in the Preferred Reporting Items for Systematic Reviews and Meta-Analyses Protocols (PRISMA-P) [13] and registered in the International Prospective Register of Systematic Reviews (PROSPERO), under the registration number CRD42019131862 [14]. This review was also based on the SR protocol published by Lima et al. [15].

The initial proposal of the study considered the inclusion of publications without delimitation of the type of molecule evaluated, and if it reduced TNF-α, as described in the systematic review protocol [15]. However, when starting the search, there was a need to restrict the type of molecule because there were studies with a range of compounds and differences between them. In addition, the different molecules could make it challenging to analyze and discuss the data. Therefore, we included in the present SR studies with amino acids, peptides and proteins, as these classes are related in structure and constitute the majority of molecules that act to reduce TNF-α evaluated in studies that assess its effect on the intestinal barrier. These adjustments were registered and updated in the SR protocol [14].

### Search strategies

Initially, preliminary equations were tested using keywords indexed in the Medical Subject Headings (MeSH), with the objective of developing search strategies with high sensitivity. The search equations (Table 1) were constructed with combinations of keywords related to the intestinal barrier, inflammation and intervention using anti-inflammatory agents. The search was performed independently by two researchers (M.S.R.L. and V.C.O.L.) in October 2021, in the electronic databases PubMed, Scopus, Web of Science (WOS), Excerpta Medica Database (EMBASE) and ScienceDirect [15].

**Table 1:** Search strategies used in each database to select articles to compose the systematic review, aiming to answer the question: What are the mechanisms of action of molecules with anti-TNF-α activity in the intestinal barrier?

Databases	Search strategies [15]
PubMed and Scopus	“intestinal mucosa” AND “Tumor Necrosis Factor-alpha” AND “Anti-inflammatory agents”
Web of Science and EMBASE	intestinal mucosa AND anti tnf-alpha
ScienceDirect	"intestinal mucosa" AND "anti tnf-alpha"

In the search in ScienceDirect and Scopus databases, the filter “Research Articles” was used.

EMBASE: Excerpta Medica Database

### Eligibility criteria

Although a more open research question was used and, consequently, broader search strategies, only original articles resulting from experimental studies that met the eligibility criteria based on the PICO strategy (Population, Intervention, Comparison/Control and Outcomes) were included in the review (Table 2).

**Table 2:** PICO strategy used to select articles to compose the systematic review, aiming to answer the question: What are the mechanisms of action of molecules with anti-TNF-α activity on the intestinal barrier?

**P**	Rats or mice of any strain, whose intestinal inflammation has been induced and diagnosed.
**I**	Therapy with an anti-inflammatory molecule (amino acid, peptide or protein).
**C**	Rats or mice of any strain, diagnosed with intestinal inflammation and not treated with an anti-inflammatory molecule; or the animal itself, before treatment.
**O**	Measurement of TNF-α or its activity and effects on the intestinal barrier of the animals.

PICO: Population, Intervention, Comparison/Control and Outcomes.

This work did not include review articles, case reports, comments, editorials, letters to the editor, theses, publications in annals of congresses; studies in humans or other animal/cell models; studies in other organs or in the context of other inflammatory diseases that do not affect the intestinal barrier; studies with other types of molecules; studies with only healthy animals as control group; studies that did not evaluate the reduction in TNF-α concentrations or its activity after treatment; studies that did not address the mechanisms of action of the molecules studied to obtain the effects found in the intestinal barrier.

### Data extraction

The articles were imported into the Mendeley Reference Manager (1.17.11), duplicates were excluded and the reading of titles and abstracts was performed following the eligibility criteria for this step [14]. Then, the full texts that went to the next step were analyzed and the studies that met the other inclusion criteria were selected. These steps were performed by M.S.R.L. and V.C.O.L., independently, and disagreements during screening were resolved by a third researcher (A.H.A.M.). The references of the articles included were also reviewed to identify studies that were not found in the search in the databases. All included studies were then reviewed for data extraction and assessment of risk of bias.

Data extraction was performed by M.S.R.L. and V.C.O.L., independently, in a previously standardized way, creating a database containing bibliographic information (author, year of publication), data on the study population (species, sex, weight, age, diet, groups, sample number), experimental model studied (disease; type of inflammation induction; dose; time, frequency, route and vehicle of administration), intervention studied (molecule studied; dose; time, frequency, route and vehicle of administration) and outcomes on TNF-α and on the intestinal barrier, in addition to the mechanisms discussed for the effects found, according to Lima et al. [15].

### Risk of bias and study quality assessment

Following the protocol by Lima et al. [15], the risk of bias assessment was performed using the SYstematic Review Center for Laboratory Animal Experimentation (SYRCLE) tool, which has ten questions about the methodology and results of the studies, to be scored as ’yes’ when there is a low risk of bias, "no" when there is a high risk of bias and "not clear" when there is uncertainty about the risk of bias, due to data not detailed in the text [16]. The evaluation was performed independently by M.S.R.L. and V.C.O.L., who underwent previous training and calibration, and Cohen’s kappa agreement coefficient, which assesses the level of agreement between researchers, ranged from 0.81 to 1.00 [17]. Differences were resolved by consulting a third researcher (A.H.A.M.).

## Results

### Selection, characteristics and quality of studies

**Fig 1:**
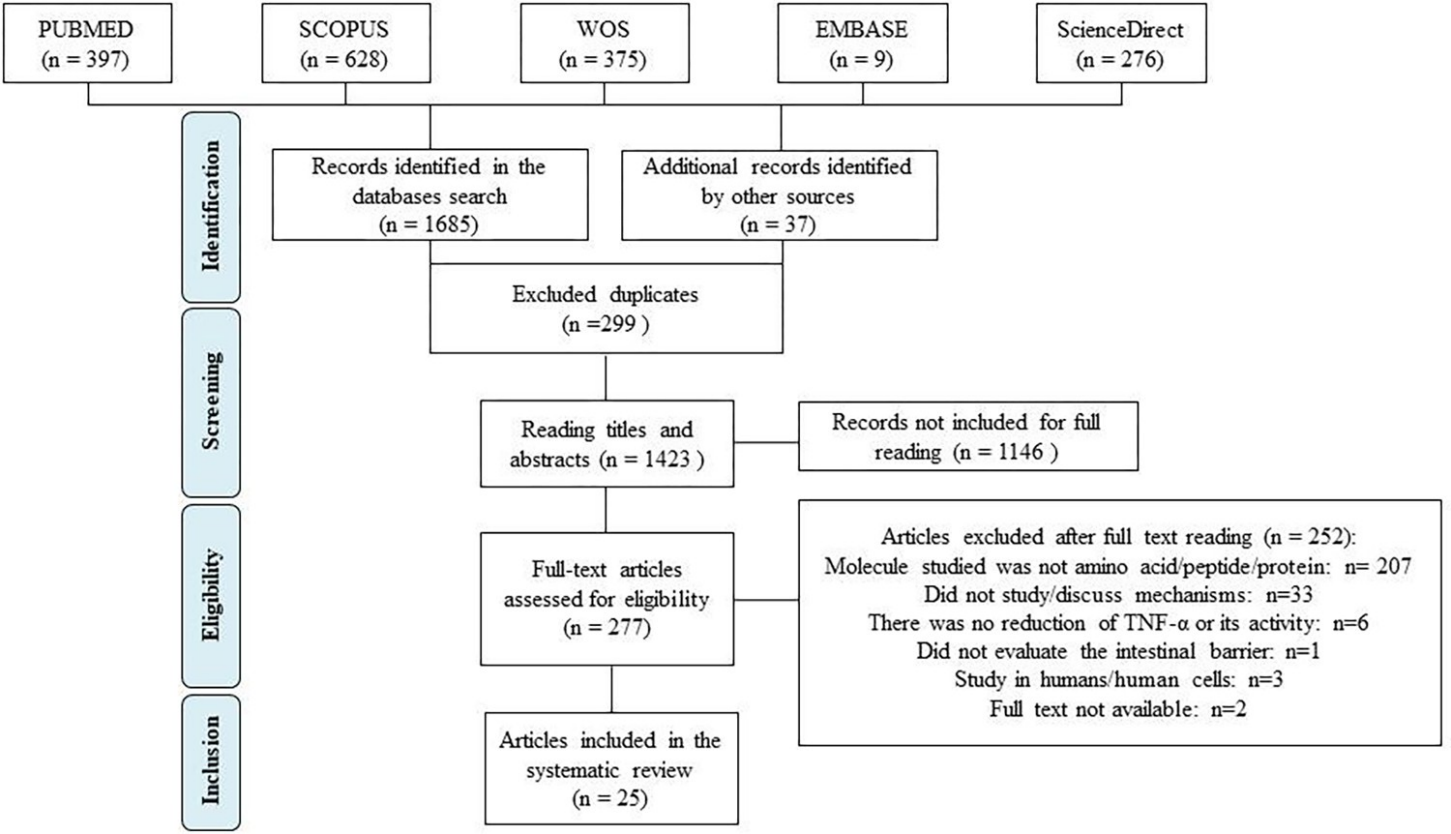
PRISMA flow diagram. Preferred Reporting Items for Systematic Reviews and Meta-Analyses Protocols (PRISMA-P) flowchart [13] of the articles included in the systematic review, aiming to answer the question: what are the mechanisms of action of molecules with anti-tnf-α activity on the intestinal barrier?

Using the search equations (Table 1) in each database, a total of 1685 records was obtained, in addition to 37 additional articles included by the manual search. After excluding the 299 duplicates, titles and abstracts were read and 1146 publications were not included for the full reading, as they did not fit the eligibility criteria considered for this step. Of the remaining 277 articles, 252 were excluded in the full text reading stage, for the following reasons: not testing amino acids, peptides or proteins (n = 207, studies with a wide variety of molecules were excluded, with emphasis on bioactive compounds of the polyphenol class, mainly curcumin), not studying or discussing the mechanisms of action of the molecules evaluated (n = 33); not mentioning TNF-α reduction or reduction/blockage of its activity (n = 6); not evaluating the effects of treatment on the intestinal barrier of the animals (n = 1); they were not studies in rats or mice, but in humans or human cells (n = 3); the full text of the publication was not available and three attempts were made to contact the authors, with no response (n = 2). Thus, 25 articles were included in the present SR (Fig 1).

Some data obtained during the extraction step were described, including the characteristics of the animals and the models of inflammation used, the description of the molecules tested and their dosages, the effects on serum/plasma and intestinal TNF-α, in addition to the main findings described in studies on the results of treatment in the intestinal barrier of the animals (Table 3).

**Table 3:** Data extracted from the studies that were selected to compose the systematic review.

**Reference**	**Animals**	**Inflammation model**	**Molecule tested**	**TNF-α analysis**	**Findings in the intestine**
**ANTI-TNF-α ANTIBODIES**					
Bhol et al. (2013) [18]	Mice C57BL/6 Males	Colitis induced by TNBS	Antibody AVX-470m 1 or 3 or 10 mg/day Oral (gavage) 2x/day, 5 days/14 days (TNBS/DSS groups)	↓ Protein expression in the colon/IHC ↓ Gene expression in the colon/qPCR	↓ Endoscopic colon score ↓ Inflammation ↓ Edema ↓ Mucosal necrosis ↓ CD68 ↓ MPO ↓ IFN-γ ↓ IL-1β ↓ IL-6 ↓ IL-12/IL-23 ↓ MMP-9
Bloemendaal et al. (2017) [19]	Mice B6.129S7-Rag1^tm1Mom/J^ (Rag1^-^/^-^) Females Mice C.B-17 SCID Females	Colitis induced by CD4+CD45RB^hi^ T cells, adoptive transfer	Murine anti-TNF IgG2c 300 μg, 2x/week Intraperitoneal 30 days Hypofucosylated murine anti-TNF human IgG1 50 μg, 2x/week Intraperitoneal 14 days	Not evaluated	↓ Severity of colitis ↓ Histological damage score ↓ IL-6 ↓ IL-1β ↓ NOS2 ↓ Severity of colitis ↓ Histological damage score ↑ Regulatory CD206 macrophages ↓ IFN-γ ↓ IL-1β
Feng e Teitelbaum (2013) [20]	Mice C57BL/6J Males 10–12 weeks	Inflammatory dysfunction induced by the use of TPN Recombinant TNF-α	Etanercept 200 μg/kg, 3x, every 48h) + 500 μg/kg, 1x, 3h before euthanasia Subcutaneous	Not evaluated	↓ Dysfunction of the epithelial barrier ↓ Permeability ↑ Occludin ↑ ZO-1 ↑ E-cadherin ↑ IκB-α ↓ MLC
Liu et al. (2007) [21]	Mice BALB/c SCID Females 8–10 weeks	Colitis induced by CD45RB^hi^CD4+ T cells, adoptive transfer	Monoclonal antibody anti-TNF 1 mg cV1q Intraperitoneal 1 x/week Early: week 0 to 8 *versus* Late: week 4 to 8	↓ Concentration in lamina propria CD4+ T cell supernatants/ ELISA	↓ Histological Colon Damage Scores ↓ CD4 ↓ F4/80 ↓ CD80 ↓ CD54 ↓ CD40 ↓ Leukocyte infiltration ↓ T CD4+ ↓ IFN-γ ↓ IL-2 ↓ IL-23p19 ↓ IL-17
**Reference**	**Animals**	**Inflammation Model**	**Molecule tested**	**TNF-α analysis**	**Findings in the intestine**
Nandi et al. (2010) [22]	Rats Sprague-Dawley Males 150-180g	Jejunoileitis induced by indomethacin	Anti-TNF-α antibody 25 or 50 μg Intraperitoneal 1x/day, 1 or 2 days	↓ Concentration in serum and small intestine homogenate supernatant/Specific Immunoassay Kit	↓ DAI score ↓ Ulceration ↓ MPO ↓ IL-1β ↓ NOx ↓ iNOS
**ANTI-IL-17 ANTIBODIES**					
Li et al. (2020) [23]	Mice BALB/c Females 18-22g	Intestinal fibrosis induced by TNBS	Anti-IL-17 antibody 200 μg/kg Tail vein injection 1 x, 8th week	↓ Serum concentration/ELISA ↓ Gene expression in the colon/RT-PCR ↓ Protein expression in colon/ Immunoblotting	↓ DAI score ↓ Intestinal fibrosis ↓ Collagen area ↓ Collagen 3 ↓ IL-17 ↓ TIMP-1 ↓ MMP-2 ↓ IL-1β ↓ TGF-β1
Song et al. (2019) [24]	Mice C57BL/6 Females 20-25g	Inflammatory bowel injury induced by body burn	Anti-IL-17A antibody 200 μg/kg Intraperitoneal 1 day before and at the time of the burn	↓ Serum concentration/ELISA ↓ Protein expression in the ileum/Western-Blot	↓ Mucosal injury severity score ↓ Intestinal permeability ↑ ZO-1 ↓ IL-17A ↓ IL-6 ↓ IL-1β ↓ T cell subtype IL-17A^+^ Vγ4^+^
**OTHER TYPES OF ANTIBODIES**					
Brasseit et al. (2016) [25]	Mice C57BL/6: Rag^-/-^ (H^+)^ 10–16 weeks	Colitis induced by adoptive transfer of CD4+CD45RB^hi^ T cells	Anti-CD4 antibodies 500 μg/animal Intraperitoneal Days 9/10, 12/13 and 15/16 post adoptive transfer Anti-TNF-α antibodies 500 μg/animal Intraperitoneal 1x/day	↓ Gene expression in the colon/ RT-PCR ↓ Serum concentration/ELISA ↓ Concentration in colon homogenate/ Multiplex assay for cytokines	↓ Clinical, macroscopic and histopathological scores ↑ Globet cell function ↑ MUC2 ↑ Proliferation of epithelial cells ↓ T CD4 ↓ Neutrophils ↓ Monocytes ↓ Macrophages(Cx3cr1^lo^) ↓ *IFN-γ* ↓ *IL-1α* ↓ *IL-1β* ↓ *IL-6* ↓ *IL-33* ↓ *TREM-1* ↓ *TNF-α* ↓ *IL-17A* ↓ *IL-17F* ↓ *CCL2* ↓ *CCL3* ↓*CXCL1* ↓ *CXCL2* ↓ *CXCL10* Preventive treatment: ↓ Histopathological score ↓ IFN-γ ↓ IL-17 ↓ IL-6 ↓ IL1-α ↓ Cxcl9 ↓ G-CSF
**Reference**	**Animals**	**Inflammation Model**	**Molecule tested**	**TNF-α analysis**	**Findings in the intestine**
Fiorucci et al. (2002) [26]	Mice BALB/c SCID e RAG-1^-/-^ Females 6–8 weeks	Colitis induced by TNBS	Anti-α1 antibody 1 mg/kg Subcutaneous 1x/day, 7/14 days	↓ Gene expression in the colon/ RT-PCR ↓ Concentration in plasma and colon culture supernatants/ELISA	↓ Histological score ↓ MPO activity ↓ IFN-γ ↓ IL-2
McDermott et al. (2014) [27]	Mice C57BL/6 Males 5–9 weeks	*Clostridium difficile* infection	Monoclonal antibody anti-GM-CSF 250 μg/injection Intraperitoneal 3x, 48h break	↓ Gene expression in the colon/RT-PCR	↓ IL-1β ↓ iNOS ↓ CXCL1 (KC) ↓ CXCL2 (MIP-2) ↓ Neutrophils ↓ SLPI
Souza et al. (2003) [28]	Rats Wistar Males 200-220g	Intestinal injury induced by mesenteric artery ischemia- reperfusion	IL-10 (1μg/animal) IL-1β (1μg/animal) Anti-IL-1ra 0.5 ml/animal Subcutaneous 60 min. after ischemia	↓ Concentration in duodenal homogenate supernatants/ELISA ↓ Serum concentration/ELISA	↓ MPO ↓ Vascular permeability ↓ Tissue hemoglobin ↓ IL-6 ↑ IL-10 ↑ IL-1β
Ungaro et al. (2009) [29]	Mice C57BL/6J 8–12 weeks	Acute colitis induced by DSS 2.5%	1A6 (anti-TLR4 antibody) 20 mg/kg Intraperitoneal Early: days 0 and 3 *versus* late: days 7 and 10	↓ Concentration in colon culture supernatants/ELISA	Early treatment: ↓ DAI score in colon ↓ CD11c^+^ ↓ CD68 ↓ CCL2 ↓ CCL20 ↓ TNF-α ↓ IL-6 Late treatment: ↑ Mucosal damage ↓ Proliferation of epithelial cells ↓ COX-2 ↓ PGE2 ↓ Ampirregulin
**OTHER TYPES OF PROTEINS**					
Hwang et al. (2020) [30]	Mice SOD1^+/-^ B6.129S7-Sod1tm1Leb/DnJ 6 weeks	Colitis induced by DSS 2%	BA SOD 20 U, with and without spores (1 x 10^7^) Oral (gavage) 1x/day, 16 days	↓ Plasma Concentration/Multiplex Assay	↓ DAI score ↓ Colon shortening ↓ Epithelial damage ↓ ROS ↑ SOD, GPx, CAT and GSH activities ↓ Neutrophils ↓ Monocytes ↓ Macrophages ↓ CD11c^+^ ↑ CD206^+^ ↓ Dendritic cells ↑ CD11b^-^CD103^+^ ↓ CD11b^+^CD103^-^ ↓ IFN-γ ↓ IL-6 ↓ IL-1β ↑ IL-10 ↓ p-p38 ↓ p-p65
**Reference**	**Animals**	**Inflammation Model**	**Molecule tested**	**TNF-α analysis**	**Findings in the intestine**
Tsuboi et al. (2007) [31]	Rats Wistar Males 180-200g	Intestinal injury induced by mesenteric artery ischemia- reperfusion	Antithrombin 30 U/kg Iv. infusion Single dose 5 min. pre-ischemia	↓ Gene expression in the intestine/RT-PCR ↓ Concentration in the ileum homogenate supernatants/ELISA	↓ Tissue injury ↓ Intraluminal hemoglobin ↓ Intraluminal protein ↓ MPO ↓ CXCL1
Xu et al. (2020) [32]	Mice C57BL/6 Males 25g 8 weeks	Colitis induced by DSS 3.5% or TNBS 2.5%	TAT-CRYAB recombinant protein 25 μg Intraperitoneal 7 days	↓ Gene expression in the colon/RT-PCR	↓ DAI score ↓ Pathological score ↓ Intestinal permeability ↓ Globet cell destruction ↑ ZO-1 ↑ E-cadherin ↑ CK20 ↑ MUC2 ↓ *IL-6* ↓ *IL-1β* ↓ *IL-8* ↓ p-p65 ↓ p-IκBα ↓ IκBα degradation
**HYBRID PEPTIDES**					
Wei et al. (2020) [11]	Mice C57BL/6 Males 6–8 weeks	Intestinal inflammation induced by LPS	C-L (Cecropin + LL37) 8 mg/kg Intraperitoneal 1 x/day, 6 days	↓ Concentration in the jejunum/ELISA	↓ DAI score ↓ Epithelial damage ↓ Chiu score ↓ MPO ↓ Neutrophil Infiltration ↓ Apoptosis ↑ TEER ↓ Taxa CD4^+^/CD8^+^ ↓ IFN-γ ↓ IL-1β ↑ ZO-1 ↑ Occludin ↓ TLR4 ↓ p-IKK-β ↓ p-NF-κB (p65) ↓ p-IκB-α
Wei et al. (2020) [33]	Mice C57BL/6 Females 4 weeks	Intestinal inflammation induced by enterohemorrhagic *E*. *coli* (EHEC)	C-L (Cecropin + LL37) 4, 8 or 16 mg/kg Intraperitoneal 1 x/day, 3 days	↓ Concentration in the jejunum/ELISA	+ Microbiota ↓ DAI score ↓ Epithelial damage ↓ Chiu score ↑ TEER ↓ MPO ↓ IFN-γ ↓ IL-6 ↓ Apoptosis ↓ MyD88 ↓ p-IKK-β ↓ p-NF-κB (p65) ↓ p-IκB-α ↑ Occludin
Zhang et al. (2019) [34]	Mice C57BL/6 Males 6–8 weeks	Intestinal inflammation induced by LPS	LTA (LL37 + Tα1) 10 mg/kg Intraperitoneal 1 x/day, 7 days	↓ Concentration in the jejunum/ELISA	↓ Epithelial damage ↓ Chiu score ↓ CD177^+^ ↓ MPO ↑ TEER ↓ Apoptosis ↓ IFN-γ ↓ IL-6 ↓ IL-1β ↑ ZO-1 ↑ Occludin ↓ p-IKK-β ↓ p-IκB-α ↓ p-NF-κB
**Reference**	**Animals**	**Inflammation Model**	**Molecule tested**	**TNF-α analysis**	**Findings in the intestine**
Yin et al. (2011) [35]	Rats Wistar Males 180 ± 10 g 10 weeks	Colitis induced by TNBS	TBCP combined with TRBCP (1:1) 25 mg/kg/day Intragastric 1 week	↓ Protein expression in the colon/IHC	↓ Inflammatory damage ↓ Macroscopic and histological scores ↓ IL-1β ↓ IL-8 ↓ MPO ↓ NO
Zhang et al. (2019) [36]	Mice C57BL/6 Males 6–8 weeks	Intestinal inflammation induced by LPS	LTP (LL37 + TP5) 10 mg/kg Intraperitoneal 1 x/ day, 7 days	↓ Concentration in the jejunum/ELISA	↓ Epithelial damage ↓ Chiu score ↓ CD177^+^ ↓ MPO ↑ TEER ↑ SOD and CAT activities ↓ IFN-γ ↓ IL-6 ↑ ZO-1 ↑ Occludin ↓ TLR4 ↓ p-AKT ↓ p-IκB-α ↓ p-NF-κB (p65)
**OTHER PEPTIDES**					
Lin et al. (2019) [37]	Mice ICR Males 4 weeks	Intestinal inflammation induced by enterotoxigenic *E*. *coli* (ETEC)	*Bombyx mori* gloverin A2 (BMGlovA2) 4 or 8 mg/kg Intraperitoneal 1 x/day, 6 days	↓ Serum concentration /ELISA ↓ Gene expression in the jejunum/RT-PCR	↓ DAI score ↑ Villus height ↓ Crypt depth ↑ ZO-1 ↓ *IL-6* ↓ *IL-1β* ↓ *TLR4* ↓ *NF-κB* ↓ *Caspase 8* ↓ *Caspase 9* ↑ *MUC1* ↑ *MUC2* ↑ *GLUT2*
Lin et al. (2021) [12]	Mice ICR	Intestinal inflammation induced by ETEC	DEFB118 (Human β-defensin 188) 4 or 8 mg/kg Intraperitoneal 1 x/day, 6 days	↓ Gene expression in the jejunum /RT-PCR	↑ Villus height ↑ ZO-1 ↓ *NF-κB* ↓ *TLR4* ↓*IL-1β* ↓ *Caspase 3* ↑ *MUC1* ↑ *SGLT-1*
Mine e Zhang (2015) [38]	Mice BALB/c Females 16-20g 6–8 weeks	Colitis induced by DSS 5%	Poly-L-lysine (L-lysine homopolymer) 10 mg/kg/day Oral (gavage) 14 days	↓ Concentration in colon homogenate supernatant/ELISA ↓ Gene expression in the colon /RT-PCR	↓ Colitis ↓ Colon shortening ↓ Total histological damage score ↓ *IL-6* ↓ *IL-17* ↓ *IL-1β* ↓ *IFN-γ*
**Reference**	**Animals**	**Inflammation Model**	**Molecule tested**	**TNF-α analysis**	**Findings in the intestine**
Sigalet et al. (2007) [39]	Rats Sprague-Dawley Males 280-300g	Ileitis and colitis induced by TNBS or DSS 5%	(1–33)-GLP-2 (recombinant human GLP-2) 50 μg/kg Subcutaneous, 2 x/day Ileitis: 0 or 2 days after TNBS Colitis: 36h after TNBS or 5 days after DSS	↓ Concentration in ileum and colon mucosal scrapings/ ELISA	↓ Histological scores ↓ Cell proliferation rate in crypts ↓ Cell apoptosis in crypts ↓ MPO ↓ IFN-γ ↓ IL-1β ↓ iNOS ↑ IL-10
Xie et al. (2019) [40]	Mice ICR Males 4 weeks	Intestinal inflammation induced by LPS	pBD129 (porcine β-defensin 129) 4 or 8 mg/kg Intraperitoneal 1 x/day, 6 days	↓ Gene expression in the jejunum/RT-PCR	↑ Villus height ↓ Crypt Depth ↓ DAO ↓ Apoptosis ↑ *Bcl-2* ↓ *Bax* ↓ *Bid* ↓ *Caspase 3* ↓ *Caspase 0* ↓ *IL-6* ↓ *IL-1β* ↑ *ZO-1* ↑ *Occludin*

AKT: protein kinase B; Anti- IL-1ra: IL-1ra antagonist (IL-1 receptor inhibitor); CAT: catalase; CK20: keratin 20; COX: cyclooxygenase; CRYAB: alpha-B-crystalline protein; DAI: disease activity index; DAO: serum diamine oxidase (intestinal permeability marker); DEFB: β-defensin; DSS: dextran sodium sulfate; EHEC: enterohemorrhagic *Escherichia coli*; ELISA: enzyme-linked immunosorbent assay; ETEC: enterotoxigenic *Escherichia coli*; GLP-2: glucagon-like peptide 2; GLUT-2: glucose transporter 2; GM-CSF: granulocyte-macrophage colony-stimulating factor; GSH: glutathione; GPx: glutathione peroxidase; H^+^: *Helicobacter typholonius* positive; ICR: Institute for Cancer Research; IFN: interferon; IHC: immunohistochemistry; IL: interleukin; iNOS: inducible nitric oxide synthase; LPS: lipopolysaccharide; MLC: myosin light chain; MMP: matrix metalloproteinase; MPO: myeloperoxidase; MUC: mucin; NO: nitric oxide; NOx: nitrite/nitrate;; PGE2: prostaglandin E2; qPCR/RTPCR: real-time polymerase chain reaction/ reverse transcription polymerase chain reaction; ROS: reactive oxygen species; SLPI: secretory leukocyte protease inhibitor; SCID: severe combined immunodeficiency; SGLT-1: sodium-glucose cotransporter 1; BA SOD: *Bacillus amyloliquefacien*s superoxide dismutase*s*; TEER: transepithelial electrical resistance; TBCP: TNF-α binding cyclic peptide; TGF-β1: Transforming growth factor beta 1; TIMP-1: tissue inhibitor of metalloproteinase-1; TLR4: toll Like receptor 4; TNF-α: tumor necrosis factor alpha; TNBS: 2,4,6-trinitrobenzenesulfonic acid; TPN: total parenteral nutrition; TRBCP: TNFR1 binding cyclic peptide; ZO-1: zonula occludens-1.

Most of the studies (n = 20) used mice, mainly of the BALB/c and C57BL/6 strains, and only five studies were with rats, mostly Wistar (n = 4). Regarding the inflammation induction model, most studies (n = 9) used dextran sodium sulfate (DSS) or 2,4,6-trinitrobenzene sulfonic acid (TNBS) to induce colitis. Many studies (n = 8) also induced intestinal inflammation through the use of lipopolysaccharide (LPS) or microorganisms, mainly *Escherichia coli*. Some used CD4+CD45RBhi T cell adoptive transfer (n = 3), ischemia and reperfusion (n = 2) or other less used ways, such as body burn (n = 1), recombinant TNF-α (n = 1) and indomethacin to induce jejunoileitis (n = 1) (Table 3).

All included studies were with peptides (n = 10) or proteins (n = 15), mainly immunoglobulins (n = 12). Due to the different nature of the molecules, this variable was used to classify the studies, facilitating the presentation and discussion of results, in: 1) anti-TNF-α antibodies, 2) anti-IL-17 antibodies, 3) other types of antibodies, 4) other types of proteins, 5) hybrid peptides and 6) other peptides.

TNF-α was evaluated in most studies by Enzyme-Linked Immunosorbent Assay (ELISA) or Reverse Transcription Polymerase Chain Reaction (RT-PCR) techniques, in serum or intestinal tissue, and the results showed a reduction in this cytokine after treatment with anti-inflammatory molecules. Only two studies did not measure TNF-α, as the molecules were antibodies, therefore they evaluated the reduction in TNF-α activity.

Regarding treatment results on intestinal parameters, studies have shown an improvement in colitis, consequently, in clinical disease scores, as well as a reduction in histological damage scores. The results also included reduction of inflammation markers, such as cytokines (e.g., IL-6, IL-1β, IFN-γ), chemokines (e.g., CCL2, CCL20), leukocyte infiltration and apoptosis markers (e.g., caspase 3). Some studies have shown an increase in anti-inflammatory components (e.g., IL-10) and increased gene/protein expression of structural and functional components (e.g., occludin, zonula occludens-1, mucins) of the intestinal barrier.

Regarding the assessment of the risk of bias in the studies, using the SYRCLE tool, no study clearly detailed the methods used to generate a random allocation of animals (Q1). Most articles presented baseline characteristics that showed pairing between animals (Q2). However, none was clear about the blinding of evaluators during allocation (Q3), random housing of animals during the experiment (Q4) and blinding of researchers in relation to the treatment received by each group (Q5).

**Fig 2:**
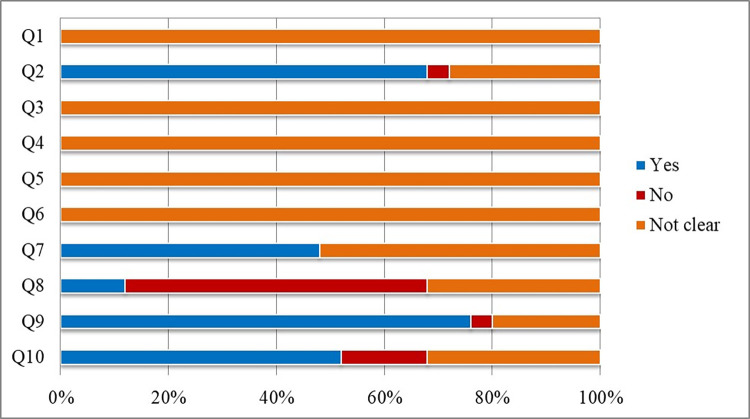
Assessment of the risk of bias of the studies included in the systematic review aiming to answer the question: What are the mechanisms of action of molecules with anti-tnf-α activity on the intestinal barrier? The articles were evaluated based on the tool of the SYstematic Review Center for Laboratory Animal Experimentation (SYRCLE), developed by Hooijmans et al. [16].

Regarding the questions related to the analysis of results, no article described whether there was random selection of animals (Q6) and nearly half reported that the result evaluator was blinded (Q7). As for the presentation of results, most studies did not adequately address incomplete outcome data (Q8), but most were also free from biased reporting of results (Q9). Regarding other potential sources of bias, most articles with a “no” answer had a conflict of interest (Q10) (Fig 2).

## Discussion

By observing the studies included in this SR (Table 3), it is clear that there was a high heterogeneity of data since different animal strains and models of intestinal inflammation were used, in addition to a wide variety of molecules tested regarding its anti-inflammatory efficacy and effects on the intestinal barrier. This heterogeneity did not allow the intended meta-analysis study to be performed, so a descriptive presentation of the data was made, classifying them based on the nature of the molecules tested.

Regarding the assessment of the risk of bias of the studies included, in many questions of the SYRCLE tool, the score attributed to the studies was equivalent to the “not clear” option, as the articles did not contain sufficient details to allow the risk of bias to be properly reported. In this regard, the authors of the tool themselves recognize that the quality of current reports of studies with animals is low, lacking many details about the conditions in which the animals are kept or about the moment of evaluating the results. For this reason, the role of this tool in increasing researchers’ awareness of the importance of improving the internal validity of animal studies [16].

Another important aspect is that the research question of this review was focused on the mechanisms of action of molecules with anti-TNF-α activity. In this regard, it is important to consider that some articles included in the review to address this issue presented different characteristics and strategies. There are articles that have as direct objective specifically to target TNF-α (mainly using antibodies). In this case, it was easier to establish mechanisms of action linked to the inhibition of production/activity of the cytokine.

In other articles, the primary objective/strategy was not specifically to target TNF-α. In these last studies, although a reduction in the production/activity of the cytokine was achieved, this is described as one of the effects, together with changes in other cytokines and molecules. Thus, the mechanisms of action of treatments associated with a decrease of production/activity of TNF-α were provided, but not all the articles included in this review have the explanation of how specifically a given treatment produces the effect on TNF-α.

### Anti-TNF-α antibodies

Anti-TNF-α therapy, performed by anti-TNF-α antibodies, is the most classically used therapy in the treatment of inflammatory diseases that affect the intestinal barrier [6]. Bhol et al. [18] studied the effects of oral administration of AVX-470m –an antibody specific against murine TNF, obtained from bovine colostrum–in three colitis models: (1) DSS-induced or (2) TNBS-induced colitis preventive model and (3) DSS-induced established colitis treatment model. The treatment was effective against colitis, with antibody delivery to the site of inflammation through minimal systemic exposure, pointed out as an advantage because monoclonal antibodies classically used to treat inflammatory bowel diseases have serious side effects as a consequence of systemic immunosuppression [41].

The results of established colitis treatment showed that the neutralization of TNF-α by the antibody generated a reduction in CD68 and myeloperoxidase (MPO) markers, indicating a reduction in macrophages and neutrophils in the colon tissue. There was also a reduction in TNF-α and TNF-α-directed pathway markers, both in relation to their protein and mRNA expression, which shows that AVX-470m inhibits not only the gene and protein expression of TNF-α, but also of several cytokines from TNF-α-directed pathways of inflammation.

In another study with experimental colitis, Liu et al. [21] induced this inflammation in mice with severe combined immunodeficiency (SCID) transfected with CD45RB^high^CD4+ T cells and treated them with an anti-TNF-α monoclonal antibody. The authors found that early and late treatments reduced inflammation, infiltration of leukocytes (CD4+ T cells and macrophages) and epithelial lesions in the colon, significantly reducing histological scores. Immunohistochemical analyzes for CD4, F4/80, CD80, CD54 and CD40 showed significantly reduced expression compared to control, suggesting that anti-TNF treatment can block leukocyte recruitment and the expression of costimulatory molecules in inflamed sites.

CD4+ T cells from lamina propria from mice treated with anti-TNF-α were also shown to secrete less TNF-α, IFN-γ and IL-2, suggesting that anti-TNF-α regulates Th1-mediated mucosal inflammation through inhibition of inflammatory cytokine secretion, without evidence of Th2 change. IL-23p19 and IL-17 mRNA expressions were significantly lower in colon tissue of mice treated with anti-TNF-α, indicating that anti-TNF-α blocks mucosal inflammation by down-regulating their expression.

Still considering anti-TNF molecules, Bloemendaal et al. [19] studied murine anti-TNF monoclonal antibodies in mice with colitis induced by adoptive transfer of CD4+CD45RB^hi^ cells. The main objective of the study was to examine the interaction between the Fc region of the anti-TNF antibody and Fcγ receptors (FcγR), and whether the absence of the Fc core fucose (which increases binding to FcγRIIIa) would increase the effectiveness of anti-TNF against colitis. The antibodies were able to reduce the severity of colitis, decreasing histological damage in the colon and mRNA expression of inflammatory cytokines, in addition to increasing regulatory CD206 macrophages. For this, the interaction between anti-TNF and FcγR was necessary. In addition, anti-TNF antibody hypofucosylation showed that the enhancement of binding to FcγRIIIa generated by this modification can make it more effective against colitis, as it significantly improved the therapeutic outcome.

Evaluating an anti-TNF-α monoclonal antibody in the treatment of indomethacin-induced jejunoiliitis, Nandi et al. [22] found that the antibody led to lower MPO activity and lower concentrations of TNF-α and IL-1β in serum and intestinal tissue in rats, compared to controls. Serum concentrations of nitrite/nitrate (NOx) and tissue expression of iNOS were also significantly lower after treatment. Thus, the inflammatory role of TNF-α was demonstrated in indomethacin-induced jejunoiliitis, suggesting that this cytokine can positively regulate other cytokines and pro-inflammatory mediators, such as the expression of iNOS, which increases the amounts of nitric oxide (NO), causing tissue damage.

Feng and Teitelbaum [20], in turn, induced intestinal inflammatory dysfunction in mice through total parenteral nutrition and evaluated treatment with Etanercept, a TNF-α antagonist consisting of a dimeric fusion protein composed of the extracellular portion of the receptor of human TNF bound to the Fc portion of human IgG 1, which binds and inactivates TNF-α [42]. Blocking TNF-α prevented epithelial barrier dysfunction and loss of junction proteins in mice submitted to TPN. NF-κB and myosin light chain kinase (MLCK) signaling were down-regulated. These results were also observed when the *TNFR1* and *TNFR2* genes were double knocked out, which showed that TNF-α is critical for epithelial barrier dysfunction associated with total parenteral nutrition, with involvement of both TNFR1 and TNFR2 receptors.

Based on the analysis of the results of studies that used anti-TNF-α antibodies, the reduction in intestinal inflammation from TNF-α blockade is the result of lower expression of inflammatory cytokines, including TNF-α, and the lower recruitment and infiltration of leukocytes in the colon, achieved through downregulation of the NF-κB pathway.

### Anti-IL-17 antibodies

Interleukin 17 (IL-17) is an inflammatory cytokine produced by Th17 lymphocytes [43]. The role of IL-17 in the pathogenesis of intestinal fibrosis in mice was evaluated by Li et al. [23], after applying an injection of IgG anti-IL-17 antibody through the tail vein. The treatment with anti-IL-17 significantly reduced intestinal fibrosis, decreased protein and mRNA expression of collagen 3, TIMP metallopeptidase inhibitor 1 (TIMP-1) and matrix metalloproteinase-2 (MMP-2), in addition to the serum concentrations of pro-fibrogenic cytokines TNF-α, IL-1β and TGF-β1. In conclusion, the authors point out that IL-17 contributed to the pathogenesis of intestinal fibrosis and that anti-IL-17 therapy could reduce this effect by down-regulating the expression of pro-fibrogenic cytokines and disrupting the MMP-TIMP balance.

Song et al. [24] also evaluated anti-IL-17A antibody therapy in mice using a model of intestinal inflammatory injury caused by body burn. When there is a burn, the integrity of the intestinal barrier can be disrupted due to local and systemic inflammatory responses. However, the neutralization of IL-17A, achieved by intraperitoneal injection with anti-IL-17A antibody one day before the burn, reduced the damage to the intestinal barrier, attenuated the alterations in the expression of zonula occludens-1 (ZO-1), prevented the increase in intestinal permeability and inhibited the increased expression of inflammatory cytokines. Antibody administration also suppressed the increase in the IL-17A+Vγ4+ T cell subtype, which was the main initial IL-17A-producing cell type in the studied model.

Although evaluated in different models of intestinal inflammation induction, the neutralization of IL-17 through antibodies generated a reduction in inflammation, acting in the reduction of inflammatory cytokines, such as IL-1β and IL-17 itself.

### Other types of antibodies

Considering the anti-inflammatory effect of other types of antibodies, Ungaro et al. [29] used the 1A6 antibody, a toll like receptor 4 (TLR4) antagonist monoclonal IgG2b, to treat inflammation in a mouse DSS-induced acute colitis model. The treatment was tested during and after colitis induction and the results showed that early treatment (days 0 and 3) attenuated acute colitis, with significantly lower concentrations of inflammatory cytokines (TNF-α and IL-6) in the colonic mucosa, compared to untreated animals. This result was attributed to the reduction of chemokines (CCL2 and CCL20) caused by the blockade of the TLR4-MD-2 complex, which led to a lower recruitment of antigen presenting cells (APCs)–especially dendritic cells and macrophages–to lamina propria, with consequent reduction in the production of inflammatory cytokines.

However, in the late treatment (days 7 and 10), there was an impairment to the mucosal healing of the animals, due to the reduction of repair mediators and intestinal homeostasis (COX-2, PGE3 and ampirregulin) because TLR4 is also important for mucosal repair, suggesting that its blockage is most effective during the early stages of intestinal inflammation.

Tool like receptors (TLRs) are transmembrane receptors that play an important role in the regulation of innate immunity by recognizing pathogen-associated molecular patterns (PAMPs), such as LPS present in the cell wall of gram-negative bacteria. The interaction of TLR4 and its MD-2 co-receptor with LPS triggers signaling cascades mediated by pathways that are or not MyD88-dependent, which lead to the translocation of the nuclear factor kappa B (NF-κB) transcription factor and the production of inflammatory cytokines [44, 45]. Thus, the activation of TLR4 leads to the production of chemokines, cytokines and antimicrobial molecules that are important in the innate immune response and in the initiation of adaptive immunity [46, 47].

Brasseit et al. [25] studied the cellular and molecular mechanisms operating in the mucosal healing process in a reversible colitis model caused by the adoptive transfer of CD4+CD45RB^hi^ T cells to lymphopenic mice colonized by *Helicobacter typhlonius*. Colitis was reversed by depleting colitogenic T cells with anti-CD4 antibodies, leading to decreased leukocyte infiltration to sites of inflammation and reduced serum and colonic TNF-α concentrations. Anti-CD4 treatment was associated with significant changes in intestinal gene expression profiles, with a rapid reduction in expression levels of several inflammatory mediators and chemokines. Colitogenic T cell depletion was also associated with restoration of colonic epithelial integrity and regeneration of the mucus layer, probably due to the recovery of globet cell function in response to mucosal healing, which was correlated with the restoration of physical separation between commensal bacteria and intestinal epithelial layer.

However, the effect of disease remission by anti-CD4 treatment cannot be attributed only to the drop in systemic and intestinal TNF-α, because when a therapeutic treatment with anti-TNF-α antibody was performed, the concentrations of other inflammatory mediators in the serum and colonic tissue from colitic mice were not affected, indicating that these mediators can compensate for the absence of TNF-α bioactivity and, therefore, mediate disease exacerbation. These discrepant findings in the literature can be attributed to the extent of intestinal inflammation present when anti-TNF-α treatment was started, as when anti-TNF-α administration was done together with the adoptive transfer of colitogenic T cells (preventive treatment), Rag^-/-^ H^+^ receptor mice were fully protected from colitis.

In the study by Souza et al. [28], intestinal injury was induced by ischemia and reperfusion of the superior mesenteric artery in Wistar rats treated with IL-1β, anti-IL-1ra (an antagonist of endogenous IL-1 receptor inhibitor) or IL-10. Reperfusion, which consists of restoring blood flow after vascular ischemia, is one of the main therapeutic goals after ischemia of a tissue or organ. However, this reperfusion can cause local and systemic inflammation, which makes it important to search for strategies that limit the damage caused by this process. The treatments mentioned were able to reduce the influx of neutrophils into the intestine, reduce vascular permeability and intestinal bleeding. IL-1β and anti-IL-1ra inhibited intestinal inflammation by suppressing the production of TNF-α and IL-6 and increased the release of IL-10 in serum and intestine.

Other results from the same study showed that the effects of IL-1 seemed to work via modulation of IL-10 production, with a consequent reduction in tissue damage and in TNF-α dependent lethality. The reduction in TNF-α production in tissue was attributed to the inhibition of neutrophil influx caused by treatments with IL-1, anti-IL-1ra and IL-10. The authors concluded that, in the studied model, TNF-α is central to the pathogenesis of injury and lethality associated with reperfusion, but both IL-1 and TNF-α seem to trigger anti-inflammatory cascades; and that IL-10 production is mainly under the control of IL-1, while TNF-α seems to activate IL-10 and another distinct molecule, not yet recognized [28].

Fiorucci et al. [26] evaluated the action of an anti-α1 monoclonal antibody in mice with TNBS-induced colitis. The α1β1 integrin, a cellular receptor for collagen/laminin, mediates inflammatory responses in contact hypersensitivity models, mediating the adhesion of leukocytes to the extracellular matrix of the mucosa. Immunoneutralization of α1 with a specific antibody protected against the development of colitis and reduced the disease severity. According to the authors, there are potential mechanisms by which anti-α1 therapy can modulate the effector phase of this disease, since the interruption of integrin/matrix interactions can affect leukocyte function at least in three levels: recruitment of leukocytes to the inflamed tissue (endothelial/leukocyte adhesion), migration of inflammatory cells through the extracellular matrix of inflamed tissue, and initiation, activation and survival of leukocytes via binding to matrix proteins. Thus, despite acting on a different receptor from the others already mentioned, the reduction in the recruitment of leukocytes is a common point present in the evaluated studies, culminating in less inflammation.

McDermott et al. [27] also observed lower numbers of leukocytes when they used a monoclonal antibody against granulocyte-macrophage colony-stimulating factor (GM-CSF) in a murine model to study the role of GM-CSF during acute colitis by *Clostridium difficile*. Treatment with anti-GM-CSF reduced the inflammatory cytokines TNF-α and IL-1β, as well as iNOS, and the expression of the neutrophil chemokines CXCL1 and CXCL2. Consistent with a decrease in the expression of neutrophil-attracting chemokines, fewer neutrophils were observed in histological sections and a reduction in gene expression of secretory leukocyte protease inhibitor (SLPI), a tissue anti-protease that protects against damage by neutrophil elastase.

### Other types of proteins

In addition to the molecules already presented, other proteins such as thrombin and its specific receptor, the protease activated receptor 1 (PAR1), act as important triggers of inflammation. Tsuboi et al. [31] evaluated the effect of antithrombin treatment on intestinal inflammation in a superior mesenteric artery ischemia-reperfusion (I-R) model. The authors observed that thrombin activity and PAR1 gene expression were increased after I-R, but the intravenous application of antithrombin before ischemia caused less injury and neutrophil infiltration in the intestine of rats. The treatment also prevented increased mRNA expression and TNF-α and CXCL1 concentrations in the ileum. The results suggest that the thrombin/PAR1 pathway has an important role in the production of cytokines during I-R and that antithrombin exerts an anti-inflammatory effect through the inhibition of inflammatory cytokines in the studied model.

Hwang et al. [30] evaluated the role of the enzyme superoxide dismutase 1 (SOD1) in a colitis model, since reactive oxygen species (ROS) play an important role in colitis and SOD is one of the superoxide dismutases responsible for the destruction of superoxide free radicals. The results showed that SOD1 produced by *Bacillus amyloliquefaciens* (BA SOD) improved disease severity, increased the activity of antioxidant enzymes, reduced the infiltration of inflammatory immune cells and, consequently, the production of inflammatory cytokines.

The authors also observed that administration of BA SOD reduced the expression of phosphorylated p38 and p65 (p-p38 and p-p65). The p38-MAPK pathway is a key regulator of cell apoptosis and this signaling promotes cell death, and the NF-κB signaling pathway activates caspase 3 and leads to apoptosis in intestinal epithelial cells. In an *in vitro* study with human colon epithelial cells transfected with SOD1 siRNA, the authors also found a reduction in p-p38 and p-p65 when the cells were treated with BA SOD, in addition to a reduction in apoptotic cells and caspase 3 and an increase in Bcl-2. Together, the results suggest that BA SOD administration suppresses oxidative stress, inflammatory response and apoptosis through inhibition of p38-MAPK-NF-κB signaling.

Xu et al. [10] evaluated the action of alpha-B-crystalline protein (CRYAB) in models of murine colitis induced by DSS and TNBS. The authors made a TAT-CRYAB recombinant protein, containing the TAT protein transduction domain, which facilitates cell penetration, to generate a cell-permeable recombinant CRYAB. Treatment with TAT-CRYAB showed anti-inflammatory action, reducing the gene expression of inflammatory cytokines and the phosphorylation of p65 and IκBα, in addition to the degradation of IκBα. Furthermore, it reduced intestinal permeability and relieved colitis in animals.

### Hybrid peptides

The results of Yin et al. [35] showed that combining a TNF-α binding cyclic peptide (TBCP) with a TNFR1 binding cyclic peptide (TRBCP) improved TNBS-induced colitis in rats. The findings showed that the peptides reduced the inflammatory response, exerting a therapeutic effect on mucosal injury. Combined treatment reduced colitis inflammation by neutralizing TNF-α and blocking TNFR1, down-regulating colonic expression of TNF-α by disrupting positive feedback and the gene expression of IL-1β and IL-8 in peritoneal macrophages. It was suggested that inhibition of these cytokines and inflammatory mediators (NO) production by blocking TNF-α signaling led to suppression of inflammatory cell infiltration, leading to attenuation of colitis. Since TBCP and TRBCP block the biological activity of TNF-α by binding to TNF and TNFR1, respectively, the combination of both peptides proved to be more efficient than the isolated peptides.

The peptide hybridization method has also been applied to antimicrobial peptides (AMP), because despite the anti-inflammatory effects of these peptides, its development faces several obstacles, mainly due to its toxicity for eukaryotic cells, impairing its clinical impact. Hybridization, however, has increased anti-inflammatory potential and stability and minimized AMP toxicity [48].

Zhang et al. [34] constructed the hybrid peptide called LTA by combining the active site of LL-37 (a small peptide derived from native AMP known to neutralize LPS) with the active site of Tα1 (a 28-amino acid peptide produced by thymic stromal cells). Tα1 has low toxicity and exhibits good immunoregulatory activity, acting through interaction with TLRs and through intracellular signaling pathways, such as NF-κB, MAPK and MyD88 pathways. However, it has relatively low anti-inflammatory activity, making hybridization an effective strategy to improve its anti-inflammatory activity and also reduce the undesirable toxic effects of native peptides.

The results of the study showed that the hybrid peptide LTA improved anti-inflammatory activity with minimal toxicity, prevented intestinal inflammation in the jejunum epithelium and leukocyte infiltration in a murine model of LPS-induced intestinal inflammation. There was a reduction in inflammatory cytokines, increased expression of tight junction proteins ZO-1 and occludin, and reduced cell permeability and apoptosis in the jejunum. The results of the quantification of NF-κB pathway proteins suggest that this is one of the ways in which LTA modulates the immune system of animals. Additional flow cytometry and molecular dynamics assays demonstrated that the interface of MD2 that binds to LPS was compared to that of LTA, suggesting that this hybrid peptide blocks the binding of LPS to the TLR4/MD2 complex.

Zhang et al. [36] evaluated another hybrid peptide, LTP, resulting from hybridization between the active center of LL-37 and thymopentin (TP5), in a model of intestinal inflammation induced by LPS. TP5 is an immunomodulator, composed of an Arg32-Tyr36 fragment derived from thymopoietin, which exerts anti-inflammatory effects and plays an important role in the maturation and differentiation of T lymphocytes. Intraperitoneal administration of the hybrid peptide led to the prevention of intestinal tissue damage, leukocyte infiltration and histological evidence of inflammation. LTP reduced inflammatory cytokines, increased superoxide dismutase and catalase activities, positively regulated the expression of tight junction proteins and decreased permeability in the jejunum, proving to be more potent than the parental peptides LL-37 and TP5. The results showed that LTP effects seem to be involved with the neutralization of LPS, with the inhibition of oxidative stress and the NF-κB signaling pathway.

Another hybrid peptide containing the functional core region of LL-37 was evaluated by Wei et al. [11], this time hybridized with the active center of cecropin A (C), called peptide C-L. This hybrid also exerted a protective effect against LPS-induced intestinal inflammation, protecting the intestinal tissue from damage by reducing leukocyte infiltration, inflammatory cytokine concentrations and cell apoptosis. Additional trials have showed that the anti-inflammatory effects of C-L are mainly attributed to its LPS neutralizing activity and antagonizing LPS-induced activation of the TLR4/MD2 complex, affecting the TLR4-NF-κB signaling pathway.

This same hybrid peptide was studied in a model of intestinal inflammation induced by infection with enterohemorrhagic *Escherichia coli* (EHEC) in mice [33]. In addition to the effects already mentioned in the previous study, treatment with C-L improved the composition of the intestinal microbiota. MyD88 expression and phosphorylation of IKK-β, NF-κB and IκB-α were reduced in the jejunum, indicating that C-L modulates intestinal inflammation through this signaling pathway.

According to Li et al. [48], AMP have been studied for their anti-inflammatory effects not only for interacting with LPS to inhibit the release of inflammatory cytokines, but also for inhibiting NF-κB translocation, decreasing the inflammatory response, which is corroborated by the findings of the studies mentioned.

### Other peptides

In addition to the hybrid peptides presented, other AMP–the defensins–were among the molecules evaluated in the studies included in the review. Defensins are an important family of host defense peptides, as are also called AMP, expressed predominantly in neutrophils and epithelial cells. β-defensins have shown a range of biological functions, such as antimicrobial and immunomodulatory, acting as important components of innate defense [32, 49].

In the study by Lin et al. [12], the action of human β-defensin 118 (DEFB188), an epididymal protein, on intestinal inflammation in mice infected with enterotoxigenic *Escherichia coli* was evaluated. Treatment with DEFB188 alleviated intestinal inflammation and epithelial injury, which was associated with reduced expression of genes involved in the inflammatory response–including *NF-κB* and *TLR4* –and with apoptosis in the small intestine, in addition to improved intestinal morphology and barrier functions.

Xie et al. [40] evaluated the preventive action of pig β-defensin 129 (pBD129) in mice that received intraperitoneal LPS. The results showed that this β-defensin also attenuated the inflammatory response and mucosal atrophy by reducing the secretion of inflammatory cytokines and the apoptosis of intestinal epithelial cells, which indicates antibacterial and anti-inflammatory properties. Thus, the authors conclude this molecule is a potential substitute for antibiotics and drugs used conventionally.

Mine and Zhang [38] studied the anti-inflammatory effects of poly-L-lysine (PL), a basic dietary polypeptide synthesized by *Streptomyces albulus* during the fermentation process, which exerts antimicrobial activity and activates calcium-sensitive receptors (CaSR) by allosteric binding. Treatment with PL in a murine model of DSS-induced colitis reduced the severity of this inflammation, prevented colon shortening and improved its morphology. In addition to the reduction in the relative expression of *TNF-α* mRNA in the colon, there was also a reduction in the relative expression of *IL-6*, *IL-17*, *IL-1β* and *IFN-γ* mRNA. Many of these effects were abrogated in animals receiving NPS-2143, an antagonist used to block CaSR activation, suggesting that PL exerts anti-inflammatory effects in the gut mediated primarily by allosteric CaSR activation. The CaSR, in the intestine, are mainly involved in the modulation of absorption and secretion, in the control of ionic transport and in the repair of the intestinal barrier.

Sigalet et al. [39] induced ileitis or colitis by TNBS and colitis by DSS in rats to study the anti-inflammatory effects of glucagon-like peptide 2 (GLP-2). In the three models, the treatment, given immediately or after the onset of inflammation, resulted in a significant improvement in mucosal inflammatory indices. The observed reduction in MPO activity suggested that neutrophil recruitment was significantly reduced by GLP-2, indicating that the treatment decreases the local production of cytokines and chemokines. The results suggest that the reduction in histological scores, MPO activity and inflammatory cytokines is due to an anti-inflammatory effect of GLP-2, which then reduces cytokine-induced apoptosis and the rate of inflammation-induced cell proliferation in crypts. In the study, GLP-2 acted through a pathway mediated by the vasoactive intestinal polypeptide (VIP), as it activated neurons in the submucosa and increased the number of neurons expressing VIP and the administration of a VIP antagonist blocked the anti-inflammatory effects of the treatment.

Lin et al. [37] evaluated an antimicrobial peptide isolated from *Bombyx mori*, called gloverin A2 (BMGlovA2). It is a peptide belonging to the cecropin family that has been shown to inhibit the growth of bacteria by binding to LPS on the surface of the bacterial membrane, which increases the permeability of this membrane. In the study, mice were treated for 6 days with BMGlovA2 and on the seventh day an intraperitoneal injection containing enterotoxigenic *Escherichia coli* (ETEC) was given. The treatment attenuated the inflammatory response and the damage to the intestinal mucosa, improving the morphology and integrity of the intestinal epithelium. The expression of genes related to apoptosis in the jejunum was reduced, while the expression of genes related to mucosal barrier functions, such as *MUC2* and *MUC1*, was increased. Thus, the authors concluded that BMGlovA2 had antibacterial and anti-inflammatory functions that make it a potential candidate to replace conventional antibiotics.

In general, the molecules belong to different groups, there is a great diversity in the evaluated outcomes and, consequently, in the pathways involved in the reduction of TNF-α and improvement of aspects related to the intestinal barrier, even within of the same class of molecules evaluated. However, most studies reaffirmed the important role of TNF-α in intestinal inflammation induced by different ways, as a cytokine that mediates an inflammatory cascade that is related to effects on the intestine, including the production of other cytokines and chemokines and recruitment of defense cells that culminate in changes in the integrity of the intestinal barrier.

**Fig 3:**
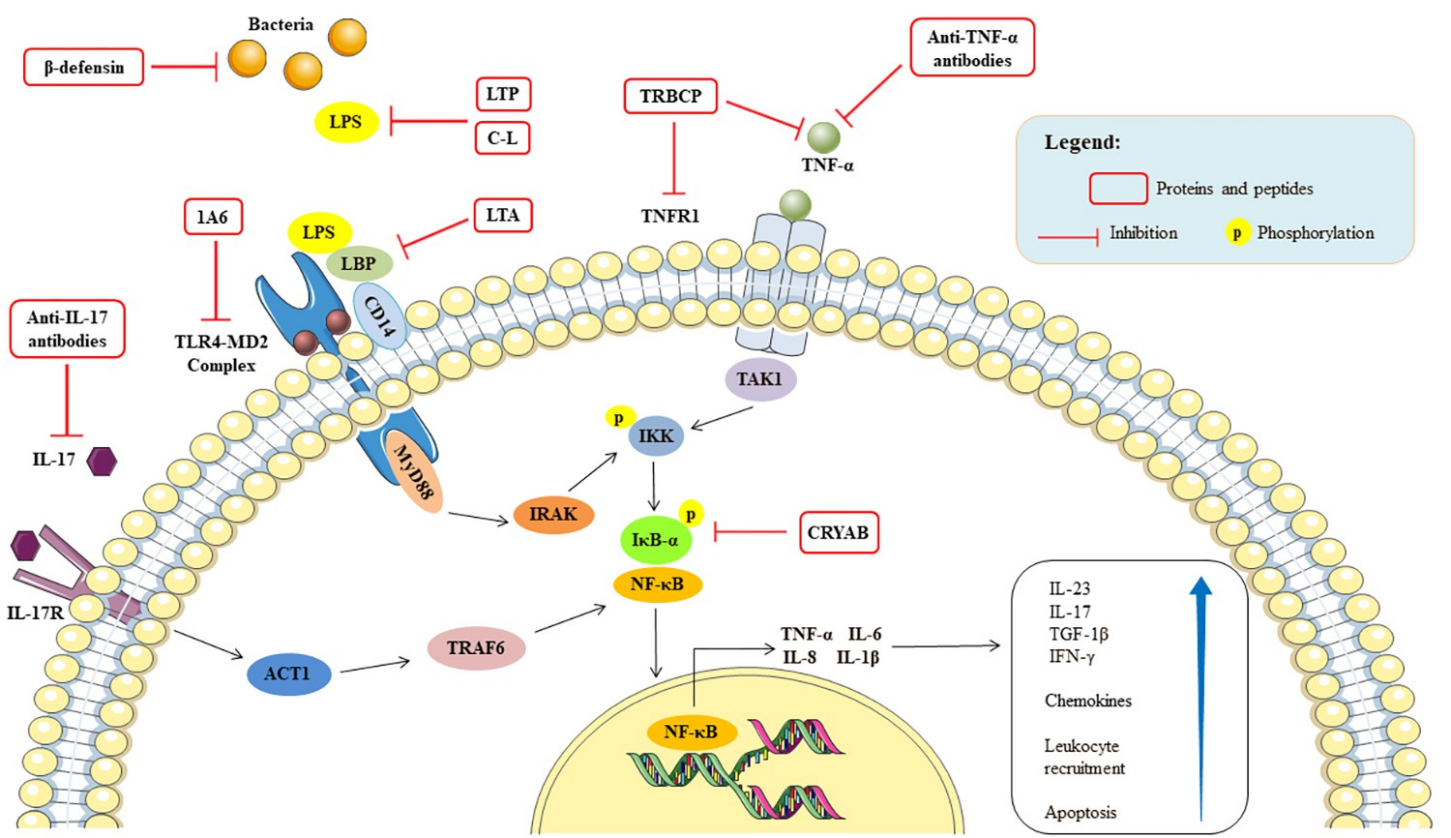
Schematic illustration of a hypothetical immune cell, presenting the main mechanisms discussed about the action of anti-inflammatory proteins and peptides with anti-TNF-alpha activity that act on the intestinal barrier. The figure was built using the Servier Medical Art images (smart.servier.com). IFN: interferon, IL: interleukin; LBP: LPS-binding protein; LPS: lipopolysaccharide; TGF: transforming growth factor; TLR4: tool like receptor 4; TNF-α: tumor necrosis factor alpha; TNFR: tumor necrosis factor alpha receptor.

The anti-inflammatory molecules studied acted by reducing TNF-α mainly by acting on the TNF-TNFR1/TNFR2 and TLR4-MD2 complex signaling pathways and, consequently, on the NF-κB signaling pathway (Fig 3). The regulation of these pathways and, therefore, of inflammation, has been shown to positively affect epithelial integrity, through the preservation of epithelial junction proteins and functional mucosal components, reduced infiltration of leukocytes, chemokines and cytokines in the intestinal tissue, which improved the inflammatory diseases, in addition to the macroscopic, histological and intestinal permeability aspects (Fig 3).

The results obtained in this review point to the potential of protein and peptide molecules that act on inflammatory pathways for medical applications, aiming to improve the inflammatory diseases that affect the intestine. The importance of this study is highlighted, since these diseases have become a global health problem with an increasing incidence in some populations.

## Supporting information

S1 Appendix Preferred Reporting Items for Systematic Reviews and Meta-Analyses Protocols (PRISMA-P) checklist. (PDF)

S2 Appendix Protocol registered in the International Prospective Register of Systematic Reviews (PROSPERO). (PDF)
