# Supplementary material for: Mechanisms of action of anti-inflammatory proteins and peptides with anti-TNF-alpha activity and their effects on the intestinal barrier: A systematic review
Source: PLoS One. 2022 Aug 8;17(8):e0270749. doi: 10.1371/journal.pone.0270749 (PMC9359527; doi:10.1371/journal.pone.0270749)
Supplement: S1 Appendix — (PDF) [file pone.0270749.s001.pdf]

Mechanisms of action of molecules with anti-TNF activity on inflammation in the intestinal barrier.

Mayara Lima, Vanessa Lima, Grasiela Piuvezam, Kesley Azevedo, Bruna Maciel, Ana Heloneida Morais

Citation

Mayara Lima, Vanessa Lima, Grasiela Piuvezam, Kesley Azevedo, Bruna Maciel, Ana Heloneida Morais. Mechanisms of action of molecules with anti-TNF activity on inflammation in the intestinal barrier.
PROSPERO 2019 CRD42019131862
Available from: https://www.crd.york.ac.uk/prospero/display_record.php?ID=CRD42019131862

Review question [1 change]

What are the mechanisms of action of molecules with anti-tnf-alpha activity on the intestinal barrier?

Context and rationale

Tumor Necrosis Factor Alpha (TNF-α) is an omnipresent inflammatory cytokine in the body, produced in response to an infection or immune injury. Its activity induces multiple responses that extend beyond its well-characterized proinflammatory actions, including several signals for cell differentiation, proliferation, and death. The TNF-α / TNF receptor (TNFR) system has several roles in normal physiological conditions and in the pathogenesis of diseases, and plays an important role in the inflammation characteristic of inflammatory bowel diseases. The intestinal epithelium is the largest and most important barrier against the external environment. It acts as a selectively permeable barrier, allowing the absorption of nutrients, electrolytes and water and maintaining an effective defense against intraluminal toxins, antigens and enteric flora. The epithelium maintains its selective barrier function through the formation of complex protein-protein networks that mechanically interconnect adjacent cells and seal the intercellular space. TNFα acts in order to disrupt different mechanisms of maintenance of this barrier, leading to damages to nutritional status and general health. Some studies seek to promote the protection or restoration of the integrity and functionality of the intestinal barrier by means of substances with anti-TNF-α activity. Understanding how these molecules act is essential for the development of new drugs with a more specific action to combat this cytokine effects. Thus, the purpose of this study is to uncover the mechanisms of action of molecules with anti-TNF-α activity on the intestinal barrier.

Searches

We will search the following electronic bibliographic databases: PubMed; ScienceDirect; Scopus; Web of Science; EMBASE. The full search strategy (see is

based on the search components “animal” and "articles"). No publication date or language restrictions will be applied. We will screen the reference lists of included studies for additional eligible studies not retrieved by our search.

Study designs to be included [1 change]

Inclusion criteria:
This review will include original articles resulting from experimental studies performed on rats and mice (in vivo and ex vivo), and will consider all study designs that allow sufficient methodological details to allow a critical evaluation of study quality.

Exclusion criteria:
Narrative reviews, case reports, comments, editorials, letters to the editor, theses, annals of congresses and articles whose methodological details are insufficient to allow a critical evaluation of the quality of the study will be excluded.

Human disease modelled
The intestine is the main organ involved in the uptake of nutrients and water, while at the same time it constitutes an essential barrier against the entry of harmful substances and pathogens from the external environment. The intestinal barrier is composed primarily of the mucosal layer, epithelial layer and underlying lamina propria. The tight junction proteins are responsible for connecting the epithelial cells of the intestine near the apical region, sealing the space between the cells and regulating the paracellular permeability by the selectivity conferring to the flow of ions, small molecules and solutes. In addition, they regulate cell polarity by preventing the diffusion of apical membrane receptors above the junctions to the basolateral membrane. This protein complex is composed of transmembrane proteins, including occludin, junctional adhesion molecules and members of the claudin family. A dysfunction in this barrier causes increased intestinal permeability, which facilitates the translocation of harmful substances and pathogens into the bloodstream. There is a relationship between the origin and / or maintenance of this dysfunction and the production of TNF-alpha. The pathophysiology of a number of diseases is associated with a dysfunctional intestinal barrier such as obesity, irritable bowel syndrome, celiac disease, food allergies, and there are studies that show possible relationships with other disorders, such as depression and schizophrenia.

Animals/population [1 change]

Inclusion criteria:
Rats or mice of both sexes; with diagnosis of intestinal inflammation at the beginning of the experiment; undergoing therapy with anti-TNF-alpha molecule (amino acids, peptides or proteins); without restriction of water or diet; studies in vivo and ex vivo.

Exclusion criteria:
Rats or mice without diagnosis of intestinal inflammation at the beginning of the experiment; evaluation of anti-TNF-alpha treatment with other types of molecules; evaluation of anti-TNF-alpha treatment for other inflammatory diseases; with restriction of water or diet; other animals models.

Intervention(s), exposure(s) [1 change]

Inclusion criteria:
Studies should include the following:
1) a measure of inflammatory process diagnosis in the intestinal mucosa mediated by tnf-alpha;
2) a measure of reduction of cytokine levels or its activity by treatment with anti-tnf-alpha molecule (amino acid, peptide or protein), regardless of the time, frequency and dosage used;
3) a measure of the result between the anti-tnf-alpha treatment and its effect on the intestinal barrier.

Exclusion criteria:
The excluded studies will be as follows:
1) articles in which there is no diagnosis of inflammatory process in the intestinal mucosa mediated by tnf-alpha;
2) articles that do not report reduction of cytokine levels by treatment with anti-tnf-alpha amino acid, peptide or protein;
3) articles that do not present data regarding the action of the anti-tnf-alpha treatment on the intestinal barrier.

Comparator(s)/control [1 change]

Inclusion criteria:
Rats or mice of both sexes; with diagnosis of intestinal inflammation at the beginning of the experiment; not undergoing therapy with anti-tnf-alpha molecule; the animal itself before treatment, will also be considered control; without restriction of water or diet; studies in vivo and ex vivo.

Exclusion criteria:
Rats or mice without diagnosis of intestinal inflammation at the beginning of the experiment; studies whose exclusive control is a healthy animal.

Other selection criteria or limitations applied
There will be no restriction of languages and year of publication.

Outcome measure(s) [1 change]

Inclusion criteria:
Serum tnf-alpha dosage and / or related parameters (eg, gene or protein expression or activity) with and without treatment with anti-tnf-alpha molecule. In addition, the effect of treatment on the intestinal barrier (protection / recovery / damage or lack of effect).

Exclusion criteria:
Studies that do not report serum tnf-alpha dosage and / or related parameters (eg, gene or protein expression or activity). Studies that do not report the effect of treatment on the intestinal barrier (protection / recovery / damage or lack of effect).

Study selection and data extraction [1 change]

Procedure for study selection
The selection of articles will be in a first moment of the reading of title and abstract, and in the second
moment by reading the articles selected in full. Two evaluators will perform the readings independently at
both times. Discrepancies will be solved with the help of a third researcher.The reference lists of the primary studies will be searched in order to find more studies.

Prioritise the exclusion criteria
Selection by title and summary:
1. It is not an original full research paper (eg, review, editorial).
2. It is not a study with mice or mice in vivo or ex vivo.
3. No molecules with anti-TNF-alpha activity were evaluated.
4. No model of inflammation of the intestinal barrier.

Full text selection:
6. Studies that do not contain a control group.
7. The studied molecule was not an amino acid, peptide or protein.
8. Studies without dosages of TNF-alpha (serum, gene or protein expression) or measurement of its activity.
9. Studies without evaluation of the effect of anti-TNF-alpha therapy on the intestinal barrier.

Methods for data extraction
Two reviewers will independently extract data from each article. We first try to extract numerical data from

tables, text or figures. If these are not reported, we will extract data from graphs using digital ruler software.
In case data are not reported or unclear, we will attempt to contact authors by e-mail (max. 2 attempts). In
case an outcome is measured at multiple time points, data from the time point where efficacy is highest will
be included.

Data to be extracted: study design
Experimental groups, control group(s) and number of animals per group.

Data to be extracted: animal model
Species, sex, weight, age and oral diet.

Data to be extracted: intervention of interest
Type of molecule administered, dose, time of administration, frequency of administration, route of administration and vehicle.

Data to be extracted: primary outcome(s)
Serum TNF-alpha (pg/mL), as well as gene expression (relative expression) and protein (mg/?).

Data to be extracted: secondary outcome(s)
Expression of barrier junction proteins, evaluation of intestinal permeability, application of epithelial damage score, histological and stereological tissue analysis.

Data to be extracted: other
1st author, year of publication, journal.

Risk of bias and/or quality assessment [1 change]
By use of SYRCLE's risk of bias tool.
Two evaluators will perform the readings independently. Discrepancies will be solved with the help of a third
researcher.
.

Strategy for data synthesis

Planned approach
The data will be presented in summary tables and in narrative form to describe the characteristics of the studies. They will be structured around the inflammation induction pathway, the nature of the molecule with anti-TNF-alpha activity administered, the dose given, the type of administration, the time of treatment, the

TNF-alpha reduction mechanism and the result obtained in the barrier (protection or recovery). We will provide summaries of the results obtained in relation to the reduction of TNF-alpha related parameters and the effect on the intestinal barrier, as well as to verify the possible mechanism by which the effect is given.

Effect measure
The effects of TNF-alpha reduction and evaluation parameters of the intestinal barrier will be evaluated by means differences.

Effect models
Because of the exploratory nature of animal studies, the random effects model will be used to account for
anticipated heterogeneity.

Heterogeneity
The heterogeneity between trial results will be evaluated using a standard X^2 test with a significance level 0,
05. To assess heterogeneity, we plan to compute the I^2 statistic, which is a quantitative measure of
inconsistency across studies. A value of 0% indicates no observed heterogeneity, whereas I^2 values of 50%
indicate a substantial level of heterogeneity. If possible, funnel plots will be used to assess the presence of
potential reporting biases. A linear regression approach will be used to evaluate funnel plot asymmetry.

Other
In the studies who will need adjust it will be adjusted accordingly.

Analysis of subgroups or subsets

Subgroup analyses
At first they are not applied, depending on the criteria of the articles that will be selected.

Sensitivity
At first they are not applied, depending on the criteria of the articles that will be selected.

Publication bias
At first they are not applied, depending on the criteria of the articles that will be selected.

Contact details for further information

Mayara Lima
mayara.lima@yahoo.com.br

Organisational affiliation of the review

Federal University of Rio Grande do Norte
https://sigaa.ufrn.br/sigaa/public/programa/portal.jsf?id=1639

Review team members and their organisational affiliations

Ms Mayara Lima. Federal University of Rio Grande do Norte
Ms Vanessa Lima. Federal University of Rio Grande do Norte
Dr Grasiela Piuvezam. Federal University of Rio Grande do Norte
Ms Kesley Azevedo. Federal University of Rio Grande do Norte
Dr Bruna Maciel. Federal University of Rio Grande do Norte
Dr Ana Heloneida Morais. Federal University of Rio Grande do Norte

Review type

Experimental animal exposure review

Anticipated or actual start date [1 change]

01 December 2020

Anticipated completion date [2 changes]

06 December 2021

Funding sources/sponsors

None

Conflicts of interest

Language

English

Country

Brazil

Stage of review [1 change]

Review Ongoing

Subject index terms status
Subject indexing assigned by CRD

Subject index terms
Animals; Inflammation; Intestines

Date of registration in PROSPERO
24 May 2019

Date of first submission
11 April 2019

Stage of review at time of this submission [2 changes]

Stage	Started	Completed
Preliminary searches	No	Yes
Piloting of the study selection process	No	Yes
Formal screening of search results against eligibility criteria	No	Yes
Data extraction	No	Yes
Risk of bias (quality) assessment	No	Yes
Data analysis	No	Yes

Revision note
The status of the final stages has been marked as complete. Database searches were updated in October 2021.

The record owner confirms that the information they have supplied for this submission is accurate and complete and they understand that deliberate provision of inaccurate information or omission of data may be construed as scientific misconduct.

The record owner confirms that they will update the status of the review when it is completed and will add publication details in due course.

Versions
24 May 2019
07 December 2020
12 February 2022
